# Launching a virtual decision lab: development and field-testing of a web-based patient decision support research platform

**DOI:** 10.1186/s12911-014-0112-8

**Published:** 2014-12-12

**Authors:** Aubri S Hoffman, Hilary A Llewellyn-Thomas, Anna N A Tosteson, Annette M O’Connor, Robert J Volk, Ivan M Tomek, Steven B Andrews, Stephen J Bartels

**Affiliations:** Dartmouth Centers for Health and Aging, Department of Community and Family Medicine, The Geisel School of Medicine at Dartmouth, 46 Centerra Parkway (HB7250), Lebanon, NH 03766 USA; Department of Community and Family Medicine, The Geisel School of Medicine at Dartmouth, One Medical Center Drive (HB7250), Hanover, NH 03755 USA; Department of Medicine, The Dartmouth Institute for Health Policy and Clinical Practice, The Geisel School of Medicine at Dartmouth, One Medical Center Drive (HB7505), Lebanon, NH 03755 USA; Department of Epidemiology, University of Ottawa, Ottawa, ON K1H 8M5 Canada; Department of General Internal Medicine, Unit 1465, The University of Texas MD Anderson Cancer Center, 1515 Holcombe Blvd, Houston, TX 77230 USA; Department of Orthopaedics, Dartmouth-Hitchcock Medical Center, Lebanon, NH 03766 USA; Collaboratory for Healthcare and Bioinformatics, The Geisel School of Medicine at Dartmouth, 46 Centerra Parkway, Suite 330, Lebanon, NH 03766 USA

**Keywords:** Decision support, Patient decision aid, Web-based, Informed patient choice, Shared decision making, Consumer health informatics, Patient-centered, User-centered, Decision technology, Osteoarthritis, Development

## Abstract

**Background:**

Over 100 trials show that patient decision aids effectively improve patients’ information comprehension and values-based decision making. However, gaps remain in our understanding of several fundamental and applied questions, particularly related to the design of interactive, personalized decision aids. This paper describes an interdisciplinary development process for, and early field testing of, a web-based patient decision support research platform, or virtual decision lab, to address these questions.

**Methods:**

An interdisciplinary stakeholder panel designed the web-based research platform with three components: a) an introduction to shared decision making, b) a web-based patient decision aid, and c) interactive data collection items. Iterative focus groups provided feedback on paper drafts and online prototypes. A field test assessed a) feasibility for using the research platform, in terms of recruitment, usage, and acceptability; and b) feasibility of using the web-based decision aid component, compared to performance of a videobooklet decision aid in clinical care.

**Results:**

This interdisciplinary, theory-based, patient-centered design approach produced a prototype for field-testing in six months. Participants (n = 126) reported that: the decision aid component was easy to use (98%), information was clear (90%), the length was appropriate (100%), it was appropriately detailed (90%), and it held their interest (97%). They spent a mean of 36 minutes using the decision aid and 100% preferred using their home/library computer. Participants scored a mean of 75% correct on the Decision Quality, Knowledge Subscale, and 74 out of 100 on the Preparation for Decision Making Scale. Completing the web-based decision aid reduced mean Decisional Conflict scores from 31.1 to 19.5 (p < 0.01).

**Conclusions:**

Combining decision science and health informatics approaches facilitated rapid development of a web-based patient decision support research platform that was feasible for use in research studies in terms of recruitment, acceptability, and usage. Within this platform, the web-based decision aid component performed comparably with the videobooklet decision aid used in clinical practice. Future studies may use this interactive research platform to study patients’ decision making processes in real-time, explore interdisciplinary approaches to designing web-based decision aids, and test strategies for tailoring decision support to meet patients’ needs and preferences.

## Background

The 2014 Cochrane Collaboration’s review of 115 trials reported that patients who used decision aids had improved knowledge of the options, more realistic expectations of potential benefits/harms, and greater clarity about what matters most to them [1]. They were also more likely to participate in decision making, and to report improved communication with their doctors.

However, several gaps remain in our understanding of the processes whereby patients engage in decision making, and of the ways in which some features of decision aids might interact with those decision-making processes [1-4]. For example, the Cochrane Collaboration’s review noted that comparisons of simple and complex decision aid formats generated mixed effects, and highlighted the need for additional studies of interactivity and presentation formats [1].

Other studies have also raised questions about the potential for targeting or tailoring patient decision aids to address patients’ preferences for different levels of information and decision support [2-10]. For example, when patients are using a decision aid, particularly delivered online, to deliberate about a tough health care decision, do monitoring and blunting coping styles persist for both information seeking and the level of engagement in active deliberation? Are there groups of patients who seek detailed risk/benefit information (e.g., “monitors” [11]) and benefit from explicit, step-wise deliberative guidance, while other groups favor a gestalt-like overview of the key facts (e.g., “blunters”) and benefit more from implicit guidance? [12,13] Would such “deliberative styles” tend to be trait-like, in that patients use the same deliberative style across a wide range of preventive, screening, acute-, and chronic-care health decisions? Or are these styles state-like, in that patients use different deliberative styles in accordance with what’s at stake in a particular health decision? For patients with chronic conditions, do their deliberative styles change over time as they gain decision-making skills? Furthermore, does the match/mismatch between the patients’ deliberative style and the type of decision aid they receive affect their information comprehension, decisional conflict, or decision quality?

One proposed approach to addressing these questions is to create a virtual decision support laboratory [3,14]. Such a laboratory could include a web-based research platform that: a) presents a patient decision aid; b) has interactive features that allow patients to self-tailor the clinical information and deliberative support they receive to meet their decision-making needs and deliberative style; and c) incorporates data collection items for research. Researchers could then observe patients’ decision-making processes in real-time, and also could vary components of the decision aid and/or its interactive features in order to test various theories and design strategies.

There are several challenges here. An interdisciplinary approach is needed to design a web-based research platform with these capabilities [4]. Several studies of web-based patient decision aids have reported positive effects on information comprehension and acceptability to patients, but studies have also reported challenges in routine use [15-29]. The recent International Patient Decision Aid Standards (IPDAS) Collaboration’s report on *Delivering Decision Aids Using the Internet* recognized the potential gains of web-based decision support, but reported mixed results about the best practices for development, evaluation, and implementation [4]. In particular, it noted the paucity of studies reporting development and evaluation approaches derived from consumer health informatics and human factors fields. Therefore, the long-term goal of this planned program of research is to build a virtual decision lab and use it to investigate both fundamental questions about the design of interactive and tailored decision aids, as well as applied questions about the effects of a match/mismatch between patients’ deliberative styles and the types of decision support they receive on decision-making outcomes.

This paper describes the development and field-testing process used to create the virtual decision lab, which had three primary objectives. The first objective was to use an interdisciplinary, theory-based, patient-centered approach to designing a web-based decision support research platform containing three components – an introduction to shared decision making, a web-based patient decision aid with interactive features, and data collection items. The second objective was to test the feasibility of using a) this web-based research platform (in terms of recruitment, acceptability, and usage) and b) the web-based decision aid component (in terms of its performance compared with the videobooklet decision aid used in clinical practice), for planned future research studies.

## Methods

**Figure 1:**
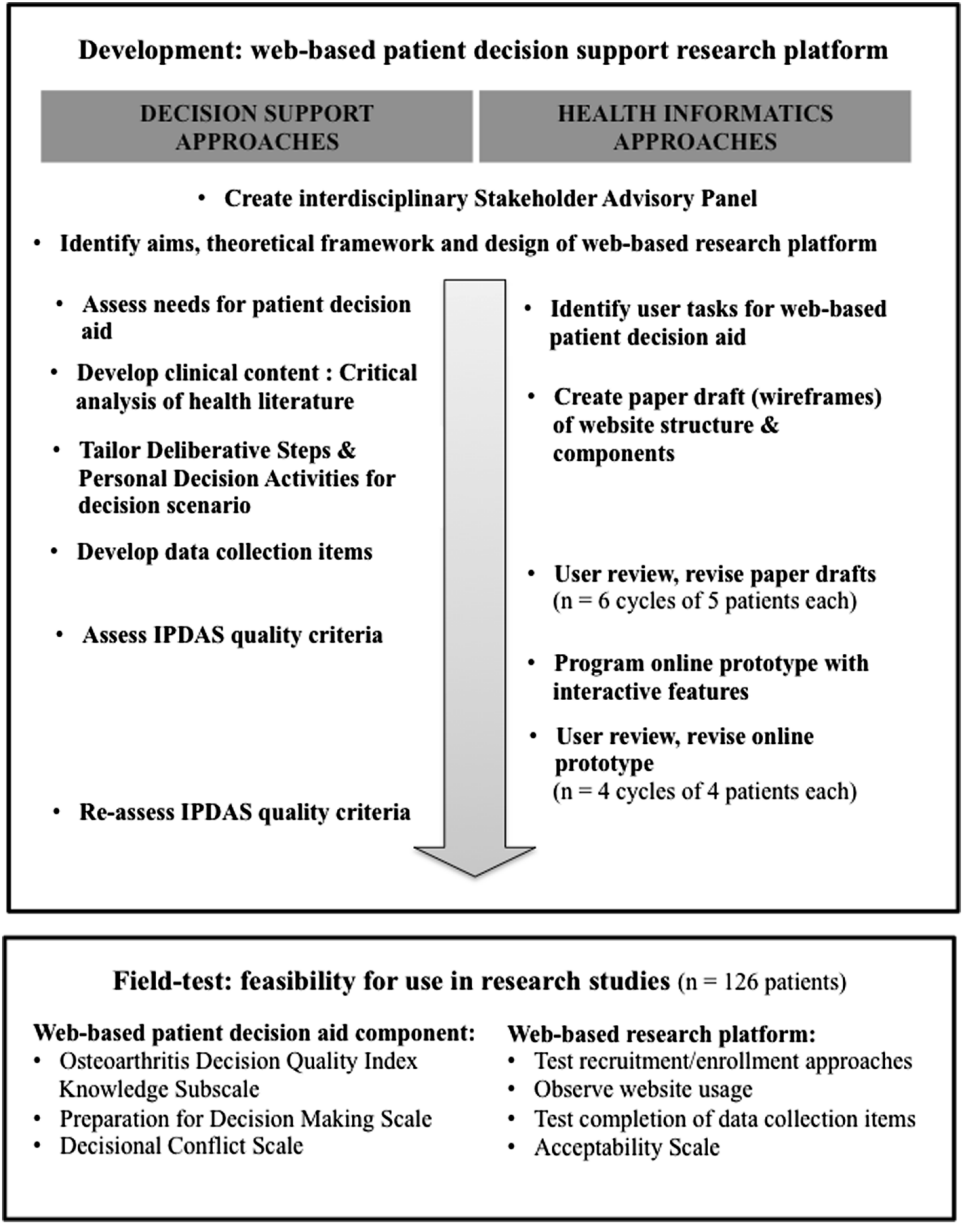
**Study design: integrating decision support and health informatics approaches for rapid-cycle development and field-testing.**

Figure 1 illustrates the study design for the development and field-testing of the web-based research platform.

### Development

To ensure that our design process addressed multiple users’ needs, we formed a stakeholder advisory panel consisting of four patients, two clinicians, two decision scientists, two decision counselors, and two health informaticians. The advisory panel selected three publications to guide development. From a decision science perspective, the Ottawa Decision Aid Development Workbook describes a protocol for theory-based, evidence-based, and patient-centered development of patients’ decision aids [30,31]. Second, the International Patients’ Decision Aid Standards Collaboration’s guidelines provide 63 quality criteria for patient decision aids: 27 criteria are related to the content of a decision aid, 30 to its development process, and 6 to the evaluation of its effectiveness [2]. Third, from a consumer health informatics perspective, the Human-Computer Interaction Handbook describes methods for identifying users’ needs and for improving the design of web-based tools by engaging users in rapid iterative cycles of design, evaluation, and redesign [32].

The panel guided development of the web-based research platform, which contained the three components: a) an introduction to the decision-making situation and baseline data collection items; b) a patient decision aid containing up-to-date clinical information and guidance in four interactive deliberative steps, with embedded process data collection items; and c) a printable Personal Decision Summary and post-decision aid data collection items. Development proceeded in three phases.

### Selecting the clinical context

Because the focus of this study was to rapidly develop a web-based research platform containing a patient decision aid, the stakeholder advisory panel sought a clinical context that did not involve a hypothetical, acute, or life-threatening decision, and for which there existed a high-quality decision aid that could provide up-to-date clinical content. From among several decision dilemmas considered, the panel selected chronic knee osteoarthritis as the clinical context for this initial study for three reasons.

First, prevalence, incidence, and surgical utilization rates for the management of chronic knee pain are high and variable. Worldwide, prevalence estimates range from 2,369 to 20,238 per 100,000 men over 45 years old, and incidence ranges from 5 to 1,263 per 100,000 person-years (21 to 27 million in the U.S.) [33-35]. Estimates are 1.5 - 2.0 times higher for women. The World Health Organization concluded that osteoarthritis is the 4^th^ leading cause of years lost due to disability [34]. Previous studies have also observed variations in surgical rates. The U.S. National Arthritis Workgroup estimated that over 300,000 total knee replacement surgeries are performed annually in the U.S., and surgical rates vary by geographical location (3-fold), sex (2-fold), and race/ethnicity (2-fold) [36-41].

Second, for many patients the choice between nonsurgical or surgical therapies for this condition is “preference-sensitive”, in that it involves considering their informed preferences about the likelihood of risks and benefits [40,41]. Mild osteoarthritis may be treated with one or a combination of pain medications, weight loss/exercise, physical therapy, etc. Patients with moderate disease may continue with non-surgical therapies and/or consider joint injections or total knee arthroscopy. For advanced osteoarthritis, multiple options are available for surgery (e.g., partial, total, bilateral knee replacements). Hence, knee osteoarthritis sufferers face repeated decisions about continuing, combining, or switching therapies.

Lastly, paper and video patient decision aids about knee osteoarthritis management have been rigorously developed and evaluated [1,31,41-44]. At the Dartmouth Center for Shared Decision Making, patients may self-refer or be referred by their clinician to view a videobooklet decision aid titled, “Treatment Choices for Knee Osteoarthritis” (**©**Health Dialog 2006) [44]. The Center monitors usage rates, as well as patients’ responses to the Osteoarthritis Decision Quality Index’s Knowledge Subscale, and the Preparation for Decision Making and Decisional Conflict scales (Table 1) [45-47].

**Table 1:** **Properties of the outcome measures used to assess feasibility of the virtual decision lab**

**Measure**	**Clinical contexts**	**Format**	**Scoring**	**Psychometric properties**
**Decision aid acceptability [** 1 **,** 62 **]**	Knee Osteoarthritis.	6 multiple choice items scored 1 “very/just right”, and 0 “somewhat/not at all” Adapted from the 10-item Ottawa Acceptability Scale. Available in English.	0-100 with higher scores indicating more acceptable.	None reported.
Assesses patients’ subjective rating of the decision aid’s ease of use, clarity of information, length, level of detail provided, ability to hold one’s interest, and satisfaction with “how the website prepared you for discussing this decision with your doctor(s)” (Adapted from the 10-item Ottawa Acceptability Scale, O’Connor 1996).				
**Osteoarthritis decision quality index’s knowledge subscale [** 1 **,** 45 **]**	Knee Osteoarthritis.	5 multiple-choice items scored 1 (correct) or 0 (incorrect). Available in English.	0-100, with higher scores indicating better comprehension.	Retest reliability ICC = 0.83. Discriminates between patients and clinicians (p < 0.001) and patients who view decision aid and patients who had usual care (p < 0.001).
Assesses patients’ objective understanding of a) which treatment is most likely to relieve pain, b) rates of improved pain, c) rates of second replacement surgery, d) rates of complications, and e) months needed for recovery.				
**Preparation for decision making scale [** 1 **,** 46 **,** 64 **-** 66 **]**	Orthopaedics, prostate cancer, breast cancer, autologous blood donation, hormone replacement therapy.	10-item version, using 5-point Likert scale from 1 “not at all” to 5 “a great deal”. Available in English French, German, Italian.	0-100, with higher scores indicating better preparation.	Alpha coefficients 0.92 to 0.96. Discriminates between people who do/do not find the decision aid helpful (p < 0.0001). Correlates with informed (r = −0.21, p < 0.01) and support (r = −0.13, p = 0.01) subscales of Decisional Conflict Scale.
Assesses patient’s perspective of how well an intervention prepared them to communicate with their physician about a decision. Includes identifying a decision, preferred role, values clarification, communication.				
**Decisional conflict scale [** 1 **,** 47 **,** 66 **]**	Osteoarthritis, disc herniation, spinal stenosis, prostate cancer, breast cancer, prenatal screening.	10-item low literacy version, using a 3-point Likert scale from 0 “yes” to 4 “no” Available in English, Spanish.	0-100, with scores below 25 associated with making a choice and scores above 37.5 associated with delaying decisions. For every unit increase, people are 59X more likely to change their mind, 23X more likely to delay decision, 5X more likely to express decisional regret, 3X more likely to fail knowledge test, and 19% more likely to blame doctor for any bad outcomes.	Alpha coefficients >0.78. Discriminates between people who make and delay decisions; effect size ranges 0.4 to 0.8. Correlates to related constructs of knowledge, regret, and discontinuance.
Assesses patients’ perceptions of uncertainty about the options, modifiable factors contributing to uncertainty, and sense of effective decision making. Includes a Leaning Scale measuring strength of treatment preference and four subscales measuring uncertainty, informed, values clarification, and support.				

### Designing the web-based research platform components

The advisory panel outlined the tasks that a series of potential users would be asked to complete, and created an initial set of paper drafts, or “wireframes”, of the following three components of the web-based research platform.

#### Introduction and baseline data collection

The website opened with a welcome and log-on page inviting eligible patients to re-review the informed consent documentation, and to confirm consent by entering their study password. No personally identifiable information was collected by the study website. Optional voice-over audio was offered for patients who preferred to hear the text read to them.

In the Introduction, a narrator described how this health care decision has multiple treatment options, and discussed how patients can, if they choose, share in the decision-making process with their doctor(s). The narrator described the decision aid as a “resource to help you prepare for a discussion with your doctor about choosing the best treatment for your knee pain”. The narrator also explained that the website would provide an overview of the key information and decision-making steps, and that they could view more detailed information and engage in interactive decision-making activities if they chose. Finally, the narrator stated that the decision aid would anonymously collect responses into a printable Personal Decision Summary that they could discuss with their family and doctor(s).

Baseline questionnaires assessed participants’ self-reported characteristics. Socio-demographic characteristics included age, sex, race, and highest level of education. Clinical characteristics included knee pain, stiffness, and function measured by the Western Ontario McMaster Universities Arthritis Index (WOMAC® 3.1) [48]. Cognitive characteristics included decisional conflict, familiarity with the decision options, and baseline treatment preference measured by the Choice Predisposition Scale [47-50].

#### The patient decision aid and decision process data collection items

Based on the Ottawa Decision Support Framework, the advisory panel structured the patient decision aid in four “deliberative steps” [30].

##### Step 1: Information comprehension

The first deliberative step helps the patient gain a clear understanding of the clinical information (i.e., the condition, the treatment options, and the likelihood of positive/negative outcomes). To design Step 1 for this web-based decision aid, the advisory panel drew on theories of situated learning, gist/verbatim comprehension, and risk communication [51-54]. First, the narrator emphasized the importance of becoming well-informed. Next, the decision aid presented up-to-date clinical information about the natural history of knee osteoarthritis, the nonsurgical options, the surgical options, and the potential risks/benefits. A systematic review had recently been completed in the annual update of the videobooklet decision aid; a team of clinicians re-reviewed and critically appraised the original articles cited in the videobooklet and the systematic review, to confirm that the clinical information was up-to-date.

The decision aid presented the clinical information at an overview level in plain language, with available audio voiceover. Patients who desired additional detail could choose interactive “More Information” links to view definitions of medical terms, anatomical diagrams, and more detailed explanations of procedures, risks, and benefits. The decision aid then provided a side-by-side summary of the treatment options and attributes. Step 1 ended with two optional Personal Decision Activities where patients could: a) self-quiz their knowledge of the key facts, and b) document questions for their doctor.

##### Step 2: Values clarification

Once well-informed, Step 2 helps the patient consider the positive and negative attributes of each of the therapeutic options, identify which attributes matter most to them personally, and clarify the desirability/undesirability they would ascribe to each option. Design of Step 2 was guided by theories of conjoint measurement, judgment heuristics, and cognitive biases [55-57]. The narrator discussed the importance of considering whether some attributes are more important than others. Narrative examples illustrated how different patients may ascribe lesser or greater importance to the different attributes of particular procedures. For example, two narratives described how patients felt differently about the trade-off between spending 1–2 hours every week in physical therapy versus needing 1–2 months off work for surgical recovery.

Finally, Step 2 presented two interactive Personal Decision Activities in which the patient could: a) rate the importance of each option’s attributes on a 0- to 5-star scale (“Not Important” to “Very Important”); and b) indicate an initially-favored option that best matched the attributes they valued most.

##### Step 3: Considering social resources

Step 3 helps patients address the logistical considerations and social influences bearing on their decision. For this decision aid, integrated information and social cognition theories guided the design [58-60]. The narrator described strategies for managing positive and negative pressures to choose a particular option, and for communicating one’s preferences with others. The narrator also emphasized the importance of considering the personal and material resources involved in undergoing one’s preferred treatment, such as the potential need for transportation to physical therapy appointments or assistance while recovering from surgery.

Step 3 presented two interactive Personal Decision Activities in which the patient could: a) list who else might be involved in the decision process and identify what the patient would like their role to be (e.g., support person, surrogate decision-maker, etc.); and b) document specific questions they had for these individuals, as well as other people involved in the decision-making, treatment, or recovery processes.

##### Step 4: Forming an Action Plan

This final deliberative step helps the patient develop a feasible strategy for moving towards an informed, values-based, actionable choice. Prospect theory and availability heuristics [61] guided the design of strategies for encouraging the patient to integrate Steps 1–3 into forming individualized action plans. The narrator discussed strategies for creating a) short-term action items to address any gaps in information, clarity, or personal support, and b) a long-term plan, such as timelines illustrating treatments, follow-up visits, and recovery stages. Step 4 ended with an optional Personal Decision Activity where patients could interactively create their personal short- and/or long-term action plans.

#### Post-decision aid data collection

To address the study’s second objective, website tracking and five post-decision aid scales assessed the feasibility of using the newly-developed web-based research platform and the web-based decision aid component in future planned research studies.

### Feasibility of the web-based research platform

To assess whether patients would use the study website and all of its components, the research platform tracked a) how many enrolled participants actually accessed the website, b) how many data collections items they responded to, and c) the time to completion. Next, to address questions about whether web-based decision support would be acceptable to patients, the platform presented the Ottawa Acceptability Scale (Table 1) [62]. For this web-based study, the scale was adapted into six multiple-choice items assessing patients’ subjective ratings of: ease of use, clarity of information, length, level of detail provided, ability to hold one’s interest, and satisfaction with “how the website prepared you for discussing this decision with your doctor(s)”.

### Feasibility of the web-based decision aid component

Before using the research platform, the advisory panel sought to confirm that the web-based decision aid component would perform comparably with the videobooklet used in clinical practice. Therefore, the study website presented the three post-decision aid scales used at the Dartmouth Center for Shared Decision Making (Table 1). The Osteoarthritis Decision Quality Index, Knowledge Subscale contains 5 multiple-choice items assessing understanding of key facts about the treatment options [45]. The interactive capabilities of the web-based research platform allowed for adaption of the paper version to provide interactive corrective feedback (i.e., if an incorrect answer was selected, the correct answer was presented). The website then presented the 11-item Preparation for Decision Making Scale and the 10-item low-literacy Decisional Conflict Scale [46,47].

In closing, the website summarized participant’s responses into their printable Personal Decision Summary, and provided links to references and related resources. It also thanked participants for participation in the study and invited them to make any final suggestions for improvement to the patient decision aid or the interactive features in an open text area.

### User review and revision cycles

Six iterative cycles of review and revision refined the paper and online prototypes. Groups of five patients walked through paper drafts of each component and were asked to comment on the wording, format, and visual layout. The drafts were revised in accordance with their comments, and iteratively presented to a new set of five patients, then revised again. Once feedback reached saturation, the advisory panel re-reviewed the optimized paper drafts and approved them for programming.

Next, informaticians and programmers of the Dartmouth Digital Decision Aid Developer Program developed the three components into an interactive web-based prototype, using Ruby on Rails (v2.3, 2009) and a password-protected MySQL database. Four focus groups of patients (n = 4 each) iteratively reviewed the prototypes online (alpha testing). Finally, the advisory panel re-appraised the patients’ decision aid component of the research platform, using the IPDAS Collaboration’s criteria, and approved the research platform for initial field-testing in the clinic (beta testing).

### Field test of feasibility for research use

#### Feasibility of the web-based research platform

The stakeholder advisory panel identified four *a priori* feasibility criteria related to website usage and acceptability. The research platform would be considered feasible if greater than 80% of eligible patients: a) could be enrolled in a project using both in-clinic and online recruitment strategies; b) could access the research website; c) would review the entire decision aid and complete all data collection items; and d) provide positive ratings on each of the six acceptability items.

#### Feasibility of the web-based decision aid component

Within the research platform, the advisory panel also identified three *a priori* criteria for considering the web-based decision aid component feasible for use in future research studies. It would be considered feasible if participants provided mean scores on the Osteoarthritis Decision Quality Index’s Knowledge Subscale, the Preparation for Decision Making Scale, and the Decisional Conflict Scale that were comparable to the mean scores observed when the videobooklet decision aid is used in clinical practice. Since this was the first use of the web-based version, blinded randomization was not used. Instead, performance of the web-based version was closely monitored and results were compared to the mean scores for the videobooklet decision aid during its clinical use over the previous two years.

The Dartmouth Committee for the Protection of Human Subjects provided ongoing ethical review and approval of this study. Participation involved a single viewing of the website between orthopaedic consultations and anonymous completion of the data collection items.

### Study participants

Study participants were recruited in two ways to ensure that the decision aid was viewed by a diverse sample of patients and to confirm that patients could be successfully enrolled in clinical settings and online. Orthopaedic surgeons at Dartmouth-Hitchcock Medical Center recruited patients who: a) were over 18 years old and able to read, write, and speak English; b) had received a diagnosis of knee osteoarthritis and were eligible for either surgical or non-surgical management; and c) were at the point of making a treatment decision. Eligible individuals were referred to the research assistant, who further explained the purpose, process, risks, and benefits of participation in the study. Verbal informed consent constituted enrollment, because the collection of signatures would involve the collection of identifiable information. Participants were offered a private room, computer, and headphones at the clinic, or a flyer with instructions for viewing the website on a personal or public computer (e.g., at a library, etc.).

In addition, collaboration with Knowledge Networks (acquired by GfK Custom Research) facilitated recruitment of a stratified sample of socio-demographically and geographically diverse participants [63]. Knowledge Networks uses address-based sampling and provides Internet access where needed to maintain a probability-based panel representing 97% of U.S. households. For this study, Knowledge Networks screened KnowledgePanel members for study eligibility criteria through a self-reported questionnaire. Potentially-eligible members were referred to the research assistant and asked to confirm that they were still in the process of considering treatment options for their knee osteoarthritis. Eligible individuals who elected to participate were enrolled, and referred to the study website.

### Data scoring and analysis

#### Baseline participant characteristics

Data analysis using Stata 10 (StataCorp® 2010) began with tabulations of the distributions of baseline characteristics of the study sample and comparison across recruitment methods. The research platform collected and scored responses to the questions about socio-demographic, clinical, and cognitive characteristics. For the Choice Predisposition Scale [49] the website scored the Leaning Scale as 0 “Unsure/No Preference” at the center; 1 to 4 in both directions for intermediate points on the scale; and 5 for the two extremes, “Strongly Prefer Surgical Treatments” at the left end and “Strongly Prefer Nonsurgical Treatments” at the right end. Scores of 4 or 5 were considered indicative of a baseline treatment preference.

#### Feasibility of the web-based research platform

The research team analyzed the feasibility of the research platform in terms of website usage and acceptability. First, the research platform tabulated the number of enrolled participants who accessed the website, and the number who completed all components and data collection items. The research team also performed content analysis on participants’ open-text suggestions for improvement. Second, for the adapted six-item Acceptability Scale [62], the website scored 0 for negative ratings (e.g., “Not at all Clear” or “Somewhat Unclear”) and 1 for positive ratings (e.g., “Clear” or “Very Clear”). The research team tabulated scores to determine whether more than 80% of respondents gave a positive response on each acceptability item, and to all six acceptability items.

#### Feasibility of the web-based decision aid component

The website automatically scored the Osteoarthritis Decision Quality Index Knowledge Subscale, the Preparation for Decision Making Scale, and the Decisional Conflict Scale according to their published scoring algorithms, (Table 1), then converted those scores to 0–100 units. For example, the 10 question-items of the baseline Decisional Conflict Scale assess patients’ perceived levels of: uncertainty (2 items), feeling informed (3 items), clarity about the personal value of the risks/benefits/side effects (2 items), and feeling supported in decision making (3 items) [47]. The website scored item-responses as either 0 “yes”, 2 “unsure”, or 4 “no”, then summed these scores, divided by 10, and multiplied by 25 to yield an individual’s score that could range from 0 (no decisional conflict) to 100 (high decisional conflict). For each scale, the analyst used unpaired *t* tests to compare the web-based decision aid’s mean scores with the videobooklet’s mean scores during clinical use over the previous two years.

Lastly, the analyst conducted sub-analyses to confirm that there were no differences in responses by recruitment method, and to assess whether participants’ baseline characteristics contributed to any observed results. Scatterplots were used to determine the appropriateness of a linear model of patient characteristics on the primary outcomes. Nonlinear distributions were transformed where possible and sensitivity analyses assessed the impact of outliers the primary outcomes. Univariate linear regression models assessed the likelihood that participants’ characteristics contributed to the results at the 5% significance level. Where associations were observed, multiple linear regression models assessed the relative contributions of each covariate.

## Results

### Development

Six cycles of iterative review and revision of the paper drafts (wireframes) resulted in a six-month development process that produced an optimized web-based patient decision aid for field testing in the clinic. The presentation of treatment options, risks, and benefits yielded the most feedback for revision. Patients recommended restructuring the presentation order, clarifying the wording of the clinical information, expanding the glossary of terms, and embedding additional “More Information” links. For the presentation of the decision support components, user feedback centered on minimizing text and adding additional “other” categories to the Personal Decision Activities. Review of the interactive features resulted in larger buttons/checkboxes and the inclusion of a status bar (e.g., “Step 3 of 4”) on the top of each page.

The advisory panel’s assessment of this optimized web-based patient decision aid prototype against the IPDAS Collaboration’s quality checklist [2] indicated that it met all 25 relevant criteria regarding the quality of the development process, and 21 of 22 criteria regarding content quality. The remaining criteria were not relevant because they referred to decisions regarding screening, the quality of testimonials, individualized risk estimates, or clinical effectiveness.

### Field test

#### Recruitment and baseline participant characteristics

In the clinic, all eligible patients within a four-month period (n = 55) were offered participation in the study; one declined to speak with the research assistant due to fatigue from a prolonged series of clinical appointments. Web-based recruitment of the socio-demographically stratified sample took one week; among 80 eligible individuals, 93% elected to participate. No differences were observed between recruitment sites other than differences in race from the stratified sampling approach (i.e., all 54 patients recruited from the clinic identified themselves as Caucasian, while nationwide web-based recruitment included 19 people from Caucasian backgrounds and 53 people from African American, Hispanic, or “other” backgrounds).

Table 2 summarizes the participants’ characteristics. Overall (n = 126), study participants were primarily female, Caucasian, younger adults with college degrees and moderate knee pain. Most participants (86%) had searched the Internet for information prior to talking with their clinician; however, few (4%) had seen the videobooklet version of the decision aid.

**Table 2:** **Study participants: socio-demographic, cognitive and clinical characteristics (N = 126)**

**Characteristic**	**n (%)**
***Socio-demographic***	
**Gender**	
Female	76 (61%)
Male	49 (39%)
**Age**	
18–64 years-old	74 (59%)
65–85 years-old	52 (41%)
**Race**	
Caucasian	72 (58%)
African American	37 (30%)
Hispanic	14 (11%)
Other	2 (1%)
**Highest education level**	
Some high school	4 (3%)
Finished high school	18 (14%)
Some college	37 (30%)
Finished college	66 (53%)
***Cognitive***	
**Decisional Conflict,** *0* **–** *100, mean (SD)*	31 (21)
**Familiarity with the decision**	
New diagnosis of osteoarthritis	8 (7%)
Have tried some nonsurgical therapies	42 (37%)
Have watched the decision aid video	4 (4%)
Have searched the Internet for information	98 (86%)
Have had a previous knee surgery	30 (26%)
***Clinical***	
**WOMAC**	
Pain, *0*–*5, mean (SD*	3.6 (2.2)
Stiffness, *0*–*5, mean (SD)*	4.0 (2.1)
Function, *0*–*5, mean (SD)*	3.6 (2.0)
Total, *0*–*100, mean (SD)*	35.0 (19.0)
**Treatment preference**	
Prefers nonsurgical therapies	80 (64%)
Unsure/No preference	19 (15%)
Prefers surgical therapies	27 (21%)

SD = Standard Deviation.

#### Feasibility of the web-based research platform

Table 3 summarizes the website usage and acceptability results. All participants (100%) preferred viewing the decision aid on a home or public computer rather than at the clinic. All participants viewed all components of the decision aid and completed all pre-/post-decisions data collection items. Mean viewing time was 36 minutes (minimum 12, maximum 90). Non-Caucasians spent more time viewing the website than Caucasians (mean = 47 versus 35 minutes, *s* = 42 versus 23, respectively; *F*_1,108_ = 4.4, *p*_*F*_ = 0.04), and individuals less than 65 years old spent less time than individuals over 65 years old (mean = 34 versus 49 minutes, *s* = 22 versus 43, respectively; *F*_1,109_ = 5.5, *p*_*F*_ = 0.02). All participants prepared a Personal Decision Summary for discussion with their doctor(s).

**Table 3:** **Feasibility of the web-based patient decision support research platform: recruitment, usage, and acceptability (N = 126)**

**Measures of Feasibility for Use in Research Studies**	**Percentage**
	**n (%)**
**Recruitment,** *enrolled/eligible*	
In clinic	54/55 (99%)
Web-based referral	74/80 (93%)
**Website Usage**	
Completion of decision aid and data collection items	126 (100%)
Time spent on website (in minutes), *mean (minimum, maximum)*	36 (12, 90)
Preference for viewing on home/public computer, compared to a computer provided at the clinic	126 (100%)
**Acceptability,** *% of patients who provided favorable ratings*	
Ease of use	124 (98%)
Clarity	114 (90%)
Appropriate length	126 (100%)
Appropriate level of detail	114 (90%)
Able to hold my interest	122 (97%)
Satisfaction with decision preparation	126 (100%)

More than 80% of participants provided positive ratings on all six acceptability items. Linear regression analyses revealed no differences across patient characteristics on the acceptability items. Open-text comments included, “I appreciate being able to re-review the information after my doctor’s visit”, “…at my own pace”, and “… in the privacy of my own home”. Multiple participants requested additional interactive features to create personalized risk estimates and treatment cost comparisons.

#### Feasibility of the web-based decision aid

Table 4 provides participants’ mean post-decision aid scores on the Osteoarthritis Decision Quality Index Knowledge Subscale, the Preparation for Decision Making Scale, and the Decisional Conflict Scale compared to the mean scores observed for patients who viewed the videobooklet decision aid at the Dartmouth Center for Shared Decision Making during the two years prior to the study (N = 243). For each of these three scales, participants who viewed the web-based decision aid reported higher mean scores than patients who viewed the videobooklet decision aid in clinical practice. The difference was not statistically significant for the Preparation for Decision Making Scale, but differences were statistically significant for the Knowledge Subscale and Decisional Conflict Scale. For the web-based version, there was also a statistically significant difference between these participants’ mean baseline and post-decision aid scores on the Decisional Conflict Scale (mean = 31.1 to 19.5, p <0.01).

**Table 4:** **Feasibility of the web-based patient decision aid: knowledge, preparation for decision making, and decisional conflict scores**

**Post-decision Aid Measures**	**Videobooklet Decision Aid used in Clinical Care**	**Web-based Decision Aid used for this Study**	***t*** **statistic**
	**(N = 243)**	**(n = 126)**	**(df = 367)**
	**mean** ***(SD)***	**mean** ***(SD)***	***p*** **value**
**Osteoarthritis Decision Quality Index’s Knowledge Subscale** *0 - 100% correct*	67% *(10%)*	75% *(11%)*	*t* = 4 *p* = *<0.001*
**Preparation for Decision Making Scale** *0 - 100*	70 *(26)*	74 *(30)*	*t* = 1 *p* = *0.18*
**Decisional Conflict Scale** *0 - 100*	15 *(14)*	19 *(15)*	*t* = 2 *p* = *0.03*

Note: Videobooklet data gathered during routine clinical use over the two years prior to the study.

SD = Standard Deviation.

df = degrees of freedom.

Linear regression analyses identified two main effects on the Decision Quality Index Knowledge Subscale’s scores and two main effects on the Preparation for Decision Making Scale’s scores. On the Knowledge Subscale, Caucasians scored higher than non-Caucasians (mean = 69.0 versus 58.0, *s* = 17.0 versus 16.6, respectively; *F*_1,118_ = 11.69, *p*_*F*_ < 0.001), and individuals who finished college scored higher than those without a degree (mean = 68 versus 60, *s* = 19.6 v 14.1, respectively; *F*_1,118_ = 6.89, *p*_*F*_ = 0.009). On the Preparation for Decision Making Scale, females reported higher scores than males (mean = 73.0 versus 61.3, *s* = 22.3 versus 24.3, respectively; *F*_1,123_ = 7.7, *p*_*F*_ = 0.006), and individuals less than 65 years old reported higher scores than those over 65 years old (mean = 72.2 versus 62.0, *s* = 21.8 versus 26.0, respectively; *F*_1,123_ = 5.7, *p*_*F*_ = 0.02). No significant interactions were observed among participant characteristics and decisional conflict scores.

## Discussion

This study demonstrates an initial interdisciplinary, patient-centered approach to developing an interactive web-based patient decision support research platform that appears feasible for studying the design and delivery of patient decision aids. During the development process, iterative patient-centered design cycles provided notable improvements to the platform’s content, language, format, and layout. Participants were successfully recruited in clinic and online, and were able to access the website, use the patient decision aid, and complete the data collection items. Positive results for usage and acceptability were observed.

Within the research platform, the interactive web-based version of the patient decision aid performed comparably to the videobooklet decision aid used in routine clinical care. On the Osteoarthritis Decision Quality Index Knowledge Subscale, participants who viewed the web-based version reported statistically significant higher mean scores, indicating improved knowledge. There was no significant difference in mean scores on the Preparation for Decision Making Scale, and these scores were similar to other published studies (mean scores between 66 and 78 across several clinical contexts) [1,47,64-67]. Mean Decisional Conflict scores were significantly higher for the web-based version than for the videobooklet; however, the post-decision aid mean for the web-based version (19.5) was below the threshold (25.0) associated with patients who proceed to making decisions [42] and reflected a significant improvement from baseline (31.0).

These results indicate that a web-based research platform supporting an interactive patient decision aid holds promise as a virtual research laboratory. However, the following limitations should be considered. First, the study sample may have contributed to a potential Type II error in terms of their high baseline familiarity. The selection of a chronic condition facilitated recruitment and minimized some confounding factors (e.g., anxiety from time-limited or life-threatening decisions), but may have constrained the ability to detect important subgroups who differ in their information comprehension, preparation for decision making, and decisional conflict. Different results may be observed with web-based research platforms and patient decision aids that focus on the first decision in a chronic condition, or on clinical situations that are acute, life threatening, or involve surrogate decision making. Furthermore, screening for low self-efficacy or minimal familiarity with the decision may facilitate tailoring the level of decision support for patients who need more/less-detailed clinical information and deliberative support.

Second, the advisory panel chose not to include video testimonials in this early version. Educational theory supports the use of videos to increase saliency, but current literature reviews regarding patients' decision support lack robust evidence that videos improve patients’ information comprehension or preparation for decision making [67-72]. Possible social matching/mismatching (by race, age, or sex) between the viewer and the individuals presented in videos could bias participants’ attitudes and treatment preferences. Notably, the web-based decision aid without testimonials performed comparably with the videobooklet version that contains testimonials. Now that this web-based decision aid has been field-tested, future studies may use this research platform to explore the purpose, structure, and optimal use of video testimonials, and other potential features of web-based patient decision aids. An interactive research program would allow such studies to proceed with large sample sizes, real-time data collection, and robust analyses [2].

Third, the IPDAS Collaborative’s report on *Delivering Decision Aids Using the Internet* distinguishes among patient decision aids that are a) posted in their original form; b) developed and evaluated using paper/video, then adapted for the Internet; and c) developed and evaluated as used on the Internet [4]. The report highlighted the challenges of combining clinical translational research and informatics approaches (e.g., how best to merge the efficacy-effectiveness-implementation pathway with the user-centered design approach), and noted that there are few published studies using such interdisciplinary approaches in a patient-centered manner. While this study designed and evaluated the decision aid as it would be used on the Internet, it falls short of a true user-centered design process, most notably in that it could not engage all users in identifying the initial purpose, goals, and tasks for the decision aid. Studies are needed to identify and evaluate best practices in engaging patients in the full user-centered design process.

Furthermore, the advisory panel elected to assess the feasibility of the research platform before engaging in more in-depth informatics and clinical studies. The intention was to ensure that participants could be successfully recruited to the research platform, use the platform, and receive comparable decision support prior to launching large randomized trials. Results indicate that this web-based research platform met the selected criteria for feasibility for research use, and subsequent studies are planned to test usability of the research platform and clinical effectiveness of the web-based patient decision aid. If the research platform is shown to be usable and effective, the virtual decision lab may then be made available to research teams to study best practices in design, optimization, dissemination, and continued improvement of web-based patient decision aids.

Fourth, the dissemination method used in this study was kept constant by providing access to a computer and headphones at the clinic. Not all clinics will have these resources; however, it is interesting to note that all participants indicated that they had access to and preferred to view the decision aid on a personal or public computer rather than at their clinicians’ office, even when a private room and computer was offered. Future research could compare dissemination methods and explore patients’ preferences for web-based decision support in and outside the clinical setting.

## Conclusions

This project illustrates one strategy for integrating decision science and informatics methods into a rapid-cycle approach for designing web-based patient decision support research platforms. An interdisciplinary stakeholder advisory panel combined with early and repeated reviews from patients meaningfully contributed to the content, format, and features of the initial prototypes. Both clinical and online recruitment methods were successful; participants completed 100% of the data collection items, and provided positive acceptability ratings. Within the research platform, the web-based decision aid performed comparably with the existing videobooklet version as used in clinical practice. Several limitations were noted that should be considered in planning future studies; however, the overall results support the feasibility of developing and using a “virtual decision lab” for patient decision support research.

### Implications

A virtual decision lab containing interactive patient decision aid(s) could be used to address a wide range of exploratory and experimental investigations [2]. A web-based research platform could be used, in real time, to address exploratory questions such as whether and how patients prefer a) to receive personalized clinical information, b) to receive lesser- or greater-detailed clinical information, or c) to proceed through the deliberative steps with lesser or greater interactive engagement at each step. Studies across clinical contexts and across time could assess whether these deliberative styles are state-like or trait-like, and whether patients’ decision support needs change as they become more skilled in the shared decision making process.

The virtual decision lab may also be used to test theory-based and interdisciplinary approaches to designing web-based patient decision aids. For example, does a user-centered approach to designing web-based tools lead to improved implementation and usage in clinical practice? Such a web-based decision lab could also be used in experimental studies testing whether important decision-making outcomes—such as information comprehension, preparation for engaging in shared decision making with one’s clinician(s)/family, or decision quality—are affected by a) the match/mismatch between patients’ deliberative styles and the design features of web-based decision support intervention, or b) the extent to which web-based decision support interventions are tailored to patients’ clinical profiles or stage of decision making.

The launch of a virtual decision research laboratory provides an opportunity to address fundamental and applied questions about how patients prefer to engage in shared decision making, and to rapidly test strategies for providing decision support interventions that meet their needs and preferences. Ultimately, these descriptive and experimental lines of investigation could contribute to the success of clinical efforts to provide targeted decision support interventions that are designed to improve patient-centered care [1,2,39,40].

## Abbreviations

IPDAS: International Patient Decision Aids Standards Collaboration
MySQL: My structured query language
WOMAC: Western Ontario McMaster Universities Arthritis Index
